# Abnormality in the Morphogenesis of Tooth Development and Relationship with Orthodontic Deformities and Treatment Approaches

**DOI:** 10.1155/2021/1183504

**Published:** 2021-11-05

**Authors:** Greta Roussanova Yordanova-Kostova, Mario Vaskov Grancharov, Gergana Diyanova Gurgurova

**Affiliations:** Department of Orthodontics, Faculty of Dental Medicine, Medical University, Sofia, Bulgaria

## Abstract

In the process of odontogenesis, a disturbance in the formation of the epithelium and mesenchyme can be observed and this can be manifested by atypical forms of dental development. Such biological phenomena with altered morphology are as follows: dens invaginatus (DI), dens evaginatus (DE), talon cusps, and double teeth (DT) or connate teeth (fusion and gemination). Patients with orthodontic anomalies who also exhibit teeth with morphogenetic disorders are presented in this article. Dens evaginatus and talon cusps pose orthodontic challenges in the treatment finishing phase. These reduce the possibility of achieving maximum intercuspidation between the lower and upper front teeth as well as poor incisor guidance. Other orthodontic challenges are as follows: the risk of occlusal trauma and periodontal loading of the antagonists and the possibility of accessory cusps to play the role of the inclined plane and lead to deviations in the closure of the lower jaw. The fused teeth can cause aesthetic and occlusal disturbances in the anterior segment. Furthermore, double teeth can lead to ectopic eruption or noneruption of adjacent teeth due to their increased crown size as is the case with one of the presented patients. This is because a double tooth occupies more space in the dental arch. If not diagnosed early, impaction of the adjacent tooth, violation of the occlusal ratios (Bolton/anterior), and exacerbation of the orthodontic deformity can be observed. The modern CBCT imaging is the best diagnostic method for identifying problems related to tooth positions or tooth germs.

## 1. Introduction

Supernumerary teeth develop themselves in addition to the normal set of teeth in dentition, and they are called under the name hyperdontia. They can be morphologically normal or abnormal in structure and characteristic, and their manifestation has no regular topography except for mesiodens. In the process of odontogenesis, a disturbance in the formation of the epithelium and mesenchyme can be observed and this can be manifested by atypical forms of dental development. The factors influencing these developmental abnormalities can be local and systemic. The altered tooth morphology sometimes looks like a tooth inserted into another tooth. This condition can be thought of as a form of hyperdontia [1]. Such biological phenomena with altered morphology are as follows: dens invaginatus (DI), dens evaginatus (DE), talon cusps, and double teeth (DT) (fusion and gemination).

Dens invaginatus (also known as dens in dente, invaginated odontoma, and dentoid in dente) is a consequence of a dysmorphogenesis, in which the partial invagination of the enamel organ at different depths is observed in the dental papilla before the calcification is finished completely. Complex variations of the internal morphology occur, divided into three types according to the Oehlers classification [2], which are illustrated and described in detail by Munir et al., Gallache et al., Lejri et al., and van der Vyver et al. [3–6]. The most severe forms are odontomoid-like and are called odontoma invaginatus. Most of the cases occur in the maxilla, where the maxillary lateral incisors are most commonly affected, followed by central incisors, premolars, canines, and molars [3].

The DI frequency cited by different authors varies from 0.3% to 10%, but this large limit is due to the different contingent of people. Cakici et al. found 1.3% DI with upper lateral involvement alone with a type I predominance of 81.25% [7]. Gündüz et al. report a 2.5% incidence of DI patients, with women being affected at a higher rate of 72% and men at 28% and again prevailing in type I with 69.8% [8]. Colak et al. establish a spread of the problem of 0.17%, with the upper lateral being affected in 4/5 of the cases and the upper canine in the rest; the bilateral form was observed only in 1/4 of patients [9]. Ceyhanli et al. cited in their study a frequency of 5.90%, regardless of gender, and type I was present in 86.6% of patients [10]. Genaro et al. in a study published in 2021 also confirm that the most common form is type I with 82.14% [11].

Priority is given to the therapeutic approach in these teeth and to the endodontic challenges with regard to the complex canal system that is often formed [4]. The gold standard for detecting dens invaginatus is radiography. The clinical diagnosis is not possible without radiographic evidence of intussusception [12]. The use of computed tomography (CBCT) is very useful in the endodontic diagnosis of complex anatomical variations [13].

Dens evaginatus (DE) is a deviation in the tooth morphology, which is manifested by an additional cusp; in fact, it is an abnormal additional tubercle. Such teeth are known as follows: Leong's premolar, evaginatus odontoma, and occlusal pearl. This protrusion consists of the enamel that covers the dentinal nucleus, where the pulp tissue is usually observed. DE is the most common morphologically altered tooth in the front segment, but can also be found on the vestibular or palatal surface, with prevalence on palatal surface of the incisors.

A talon cusp is a rare formation that presents a convex thin structure originating from the cervical region of the palatal or vestibular surface of the front teeth. The location of this tubercle is more often palatal [14]. The talon cusp overlaps with DE, composed of enamel, dentin, and varying amounts of pulp tissue. Its cited frequency is from 0.6% to 7.7% in different nations [15].

The double tooth (fusion and gemination) anomaly can develop during morphodifferentiation of dental germs due to aberration in the development of both the ectoderm and the mesoderm. Gemination is a process in which a bifid crown with one root and a common canal is developed by an invagination from one tooth. In this anomaly, the tooth is defined as a single tooth. The fused teeth occur in the process of merging two teeth into one at a certain period of embryonic development so that the fusion can be at different levels (at the enamel-dentin or dentin-cementum level). There is actually hyperdontia in the dentition. Fusion is a rare phenomenon with a total prevalence of approximately 0.5% in deciduous teeth and 0.1% in permanent dentition [16–18]. The clinical manifestation of gemination teeth is observed as the accumulation of teeth in the arch, while the fused teeth are most often associated with ectopic eruption.

The purpose of this article is to analyse the orthodontic deformity associated with the manifestation of these phenomena, the case studies with an emphasis on clinical and radiographic findings, and the diagnosis and the approaches to solving them.

We present several clinical cases of patients with orthodontic deformities and the presence of teeth with disorders in morphogenesis. Their radiological and photo documentation is used for case analysis. Prior to the diagnosis and treatment of patients, they filled in an informed consent form for the use of their data for scientific purposes without disclosing their identity. The author's team received a positive decision from the Ethics Committee “Kenimus” 2121/06.07.21 to conduct a study among orthodontic patients treated by the authors.

## 2. Clinical Case 1

**Figure 1:**
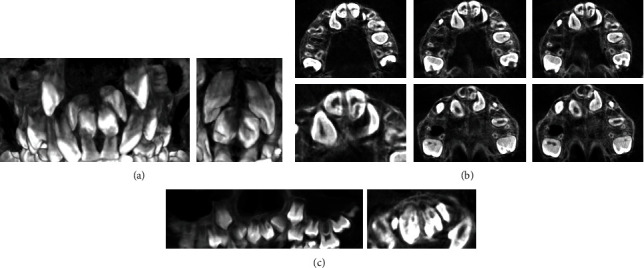
CBCT examination: (a) vestibular and palatal views, (b) transverse sections, and (с) frontal sections.

We present a case of an 8.5-year-old patient with persistent upper temporary central incisors and erupted permanent lateral incisors. On initial examination and available OPG, hyperdontia of the upper central incisors was found, with well positioned left and right incisors. The supernumerary left and right incisors are rotated and located perpendicular to the normal incisor axes. In order to make an objective assessment and choose which pair of incisors to be surgically extracted to alleviate the accumulation, a CBCT examination was ordered (Figure 1).

Based on this study, it was found that the retained central incisors, in a normal and favourable eruption position, have disturbed morphogenesis and are diagnosed with dens in dente. In the left incisor, the palatal super cusp is more pronounced, but the intussusception is determined by type II, while in the right incisor, the intussusception is of type IIIB passing through the entire root length and ending as a separate canal to the level of the not yet formed apex.

The apical development of both incisors with intussusception is incomplete. The disturbed morphology of the impacted incisors limits our choice to choose them as main central incisors, although they have a favourable position.

The orthodontic plan and treatment include the following: extraction of persistent temporary incisors and surgical removal of impacted incisors with intussusception; waiting for a 6-month recovery period; and surgical exposure and orthodontic traction of impacted upper central incisors that are ectopically embedded.

**Figure 2:**
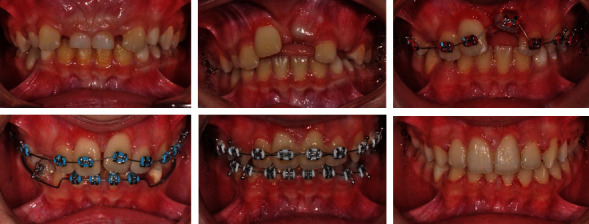
Treatment progress.

After extraction of the temporary and redundant incisors, the two ectopically located incisors spontaneously changed their position and erupted (Figure 2). Orthodontic treatment with surgical support moved the incisors and levelled the two teeth rows. After the orthodontic treatment, the patient has a fully functional and levelled dentition, with very good aesthetics and a smile.

## 3. Clinical Case 2

We present a case of a 16-year-old patient with a normal occlusal relationship at the molars but a crossbite of the right upper lateral incisor and lower canine. Left upper and lower second bicuspids were also in a crossbite. There was an edge-to-edge relationship of upper and lower left lateral incisors. Dens evaginatus was found on the upper laterals after clinical examination. These can be described as resembling the crown shape of an upper premolar with a well-defined palatal accessory tubercle with a height approaching the level of the cutting edge, as well as occlusal fissures that are coloured and predictive for carious lesions.

**Figure 3:**
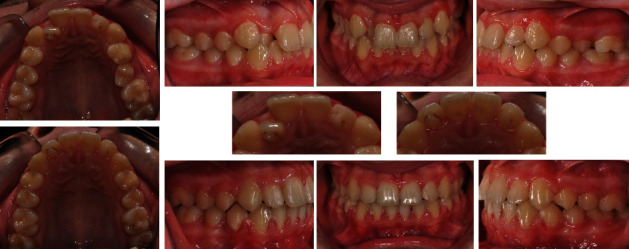
Before and after treatment: reduction of the dens evaginatus.

The upper right lateral incisor was moved out of the crossbite; however, an excessive amount of labial movement was required due to the existence of dens invaginatus (Figure 3).

The new positioning of the lateral results in a sagittal distance due to its more significant size, which disrupts the new occlusal proportions. Vestibular tipping of the central incisors is also required to compensate for the new lateral position. The new position of all upper incisors is slightly protruding because the central incisors follow the level of the laterals, which require greater vestibular inclination to level their larger labio-palatal dimension.

In the course of treatment, selective abrasion of laterals (hypertubercle) and topical fluoride application of the unveiled tooth surface were performed. In the course of the treatment, perfect white-pink aesthetics was achieved as well as normal overlap.

## 4. Clinical Case 3

**Figure 4:**
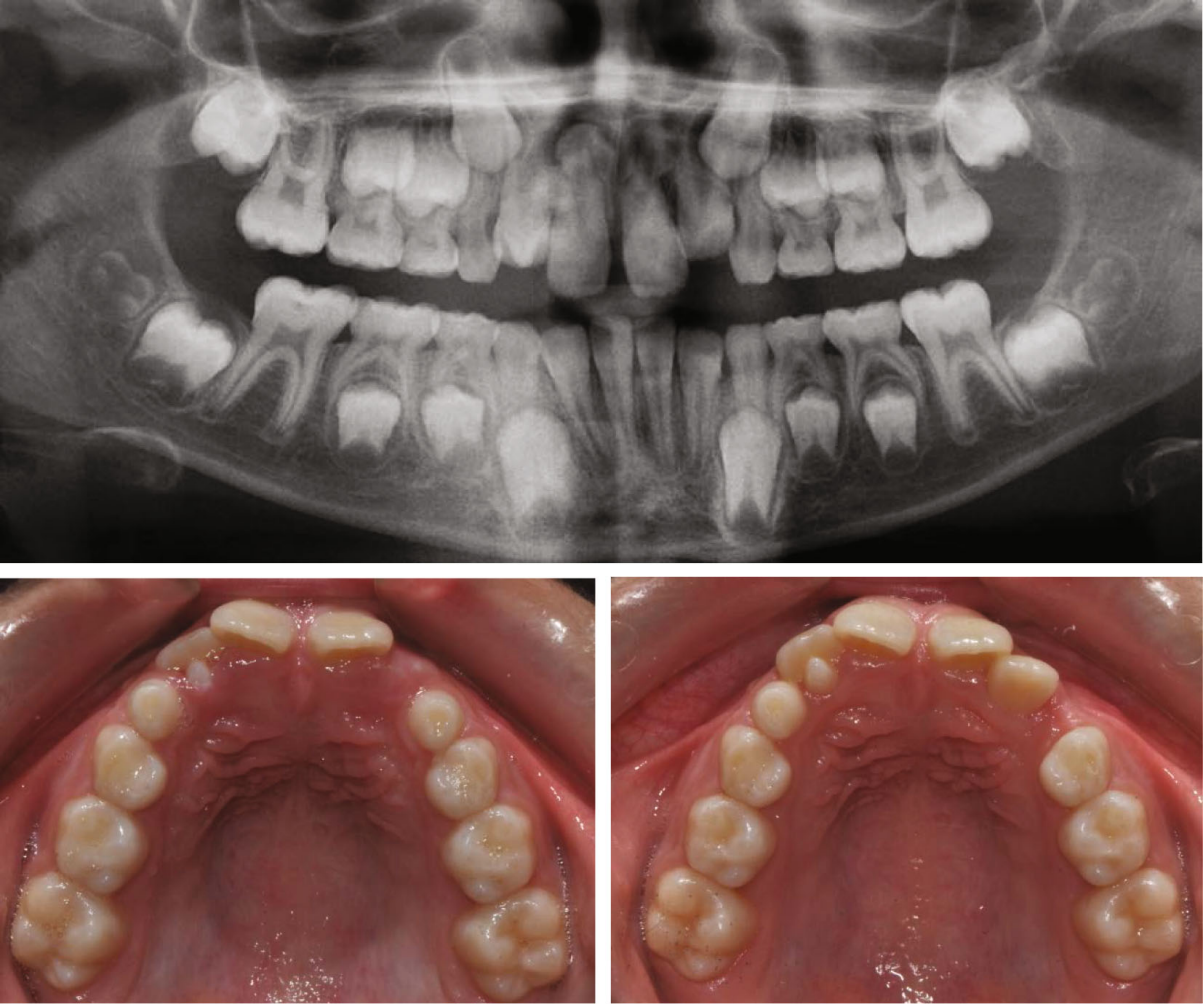
X-ray image and intraoral picture from the first clinical examination and 4 months later.

We report an 8.5-year-old patient with tooth 12 with a talon cusp and mesiodens in an in situ in verso position. At the first clinical examination, a conical formation tangential to the upper right lateral was observed. On the available OPG, mesiodens, located between the two central incisors in greater proximity to the right incisor with the inverted crown-root direction, was found. The X-ray image of tooth 12 showed the superimposition of another spike-shaped image, which completely overlapped the neighbouring tooth. Our first guess was about the second mesiodens, which erupted palatally in the immediate vicinity of the right lateral (Figure 4). We needed solid evidence of the second mesiodens because its position was not traditional.

**Figure 5:**
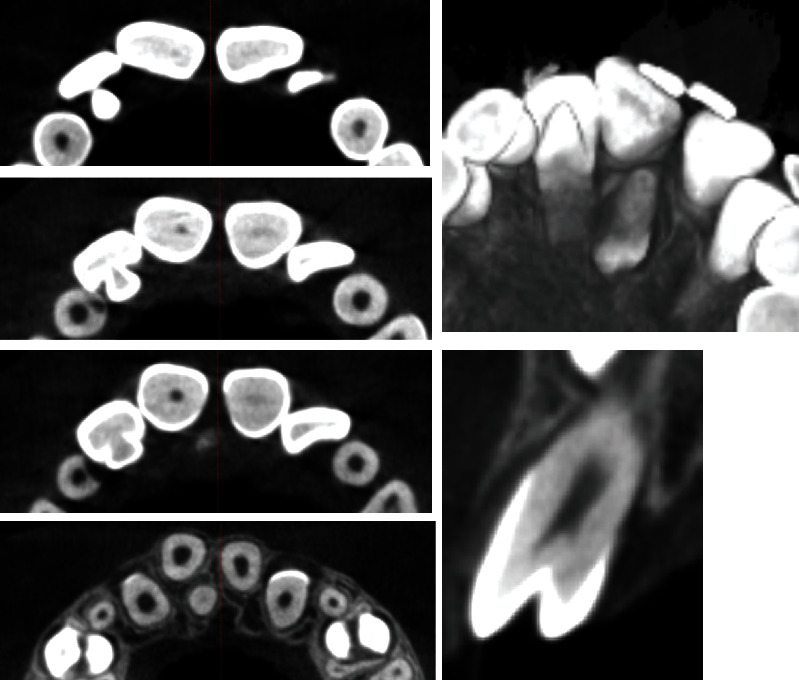
CBCT examination and transverse sections.

CBCT examination for the exact localization of supernumerary teeth (mesiodens) was ordered (Figure 5). It became clear from this study that the upper right lateral has a talon cusp, a spiked additional tip with a height equal to the crown of the lateral, whose structure is a dense layer of enamel, dentin, and only a small pulp horn branch of the pulp of the lateral.

The detailed X-ray finding changed the intentions for immediate extraction of “palatally localized mesiodens.” The talon cusp disrupts the occlusal relations between the two arches in the frontal area. The patient was kept under observation, and after a few months, the entire clinical crown of tooth 12 with the palatal talon cusp was clinically visible. The orthodontic treatment had the following stages: waiting for the apical development of the incisors to be completed; surgical removal of mesiodens; orthodontic levelling of the two dental arches; therapeutic endodontic treatment of tooth 12 and talon cusp abrasion; and adjustment of occlusal ratios and retention.

## 5. Clinical Case 4

A patient was diagnosed during a routine examination during the period of early mixed dentition with double teeth. The established anomaly was followed in its development in order to choose an appropriate time for the surgical intervention so as not to damage the root development of the adjacent teeth.

**Figure 6:**
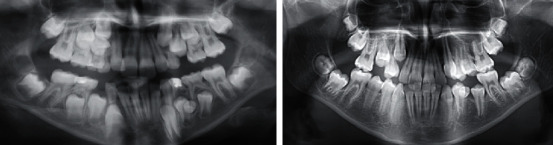
X-ray from the first clinical examination and 2 years later.

A follow-up with subsequent X-ray ascertained a double tooth (Figure 6). This was a fused tooth number 34 (lower left first premolar with an accessory crown and root). The fused supernumerary tooth is located distally in the direction of the second premolar. The adherent accessory crown has its own developed thin root, which merges with a common pulp chamber with the root of the main tooth and is inseparable from each other.

**Figure 7:**
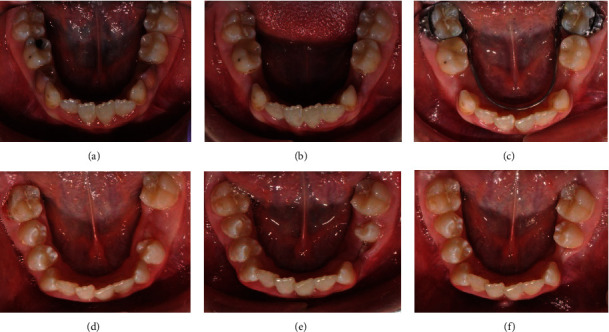
Development of the lower dental arch. (a) Initial state. (b) After extraction of temporary first molars. (c) Placed lingual arch. (d) Eruption of the lower right first and second premolars and left first premolar—double tooth. (e) Spontaneous eruption of the lower left second premolar, after extraction of tooth 34. (f) Complete eruption of the lower left second premolar.

The orthodontic treatment plan included the following: extraction of double tooth 34, preservation of the perimeter of the lower dental arch, levelling of the two dental arches, and normalization of the occlusion (Figure 7). The extraction should not lead to a shortening of the lower dental arch perimeter because the bilateral first molar relationships tended to class II. For this purpose, a lingual arch was placed to preserve the perimeter of the arch, and consequently, extraction of temporary lower left first and second milk molars was performed in order to create space for either the eruption or the extraction of the fused tooth.

**Figure 8:**
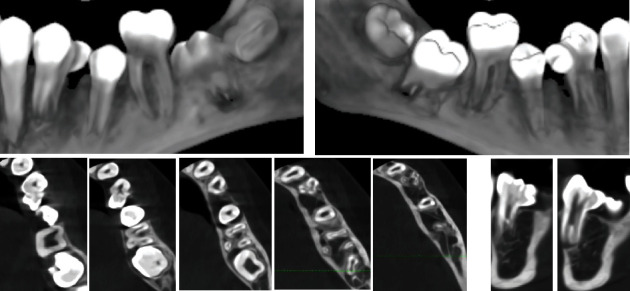
CBCT: 3D vestibular and lingual view and transverse sections.

Finally, the decision to extract double teeth was made after consultation with a maxillofacial surgeon and endodontist, who confirmed that it is not possible to guarantee sealing during devitalisation of the tooth and resection of the accessory crown. This was unfavourable due to the root and pulp morphology of the tooth (Figure 8). This solution disturbed the balance of the perimeter between the lower and upper dental arches, but the advantage was the presence of a germ of the lower left third molar, which after mesialization of the second premolar and both molars will move forward in the arch and compensate for extraction. Successful treatment requires good planning of the most appropriate time for each stage. After treatment, the patient exhibited a normal occlusal relationship and functional balance.

## 6. Discussion

Dens evaginatus and talon cusps can be major orthodontic challenges in the treatment finishing phase. We are faced with the impossibility of achieving maximum intercuspidation between the lower and upper front teeth, the inability to provide good incisive guidance, the real danger of occlusal trauma and periodontal loading of antagonists, and the possibility of accessory cusps to play the role of the inclined plane and lead to deviations of the lower jaw as well as other individual variations. The possible clinical solutions are the gradual and periodic abrasion of the tubercle and the application of topical fluoride; then, the next radical step is the removal of the tubercle together with the extirpation of the pulp and endodontic therapy of the root canals.

According to Oehlers [2], dens invaginatus is classified into three types. Maxillary lateral incisors were more affected than central incisors. Kfir et al. describe that unique clinical morphological characteristics were observed in 88% of the teeth that exhibited radiographic evidence of dens invaginatus [19]. Type II and type III are difficult to treat in endodontic therapy, and many have incompletely formed roots [20]. Therapeutic treatment of carious lesions and their complications in the intussusception area requires a high level of professionalism and endodontic skills [4–6, 20–22]. An early diagnosis of these lesions is crucial as they may adversely affect any planned orthodontic treatment, and therefore, an assessment of the prognosis of these lesions is required before starting orthodontic treatment [23]. The diagnosis of these formations usually occurs when patients seek specialist treatment for an acute therapeutic process and main orthodontic deformity or during routine examinations. The only field of paediatric dentistry in which radiography is mandatory and common practice is orthodontics. In clinical case 1 presented, the CBCT examination provided a three-dimensional representation of the intussuscepted tooth, which allowed the diagnosis and determination of the severity of the problem. The nature of the invagination and its connection to the main root canal of the tooth were assessed [24]. It is important to take proper measures according to the type and degree of dens in dente to preserve the diseased tooth as much as possible [25]. The condition of the dens invaginatus was discovered in the diagnostic phase of orthodontic treatment, which made it possible to make the most favourable decision for treatment, namely, as follows: to extract persistent temporary central incisors; to extract permanent central incisors with a favourable position, but with dens in dente and an undeveloped apex; to perform orthodontic traction for introduction into the arch of well-formed morphologically, but in an unfavourable position, central incisors. The knowledge of the phenomenon of dens invaginatus and the possibilities of 3D images made it possible to make the best long-term decision for the development of dentition and orthodontic treatment of the patient.

In the patient from clinical case 3, the talon cusp is very similar to puncture-like mesiodens. The clinical crown of tooth 12 at the first examination did not completely erupt, which does not allow diagnosing of the talon cusp only by intraoral examination. The most exact differential diagnosis is made by CBCT. The X-ray examination gives a complete picture of the structure and location of the tooth germs and the thickness of the enamel layer in the affected tooth, which determines the choice of treatment: only abrasion of the talon cusp or endodontic treatment and removal of the tubercle.

The fused teeth can cause aesthetic disturbances in the anterior segment, including other occlusal disturbances and crowding of teeth or even ectopic eruption due to increased crown size (two crowns).

After CBCT examination, a differential diagnosis was made and it was found that the patient in question has hyperdontia of the lower left premolar and a process of fusion between the normal and excess teeth (fused teeth) at the level of dentin-cementum. The additional tooth had a crown located proximal-distal and thinly attached to the main root. It occupied space in the dental arch and would make the eruption of the second premolar difficult. If the diagnosis is not made early, it is possible to break the occlusal relationship and worsen the orthodontic deformity.

The rare manifestation of these dental phenomena does not allow every orthodontist to gain experience in these matters, so each new case report and analysis of approaches in solving it enriches the orthodontic community with new clinical experience.

## 7. Conclusion

The orthodontic abnormalities are strictly individual and thus extremely variable; there are no identical patients and dentition. Therefore, it is necessary to know the anomalies associated with omitted tooth morphogenesis and the impact of this on orthodontic anomalies. The modern CBCT imaging is the best diagnostic method for identifying problems related to tooth or tooth germ positions. Patients with abnormalities in the development of teeth require careful diagnosis and planning. Therefore, it is important to know the different clinical and radiological signs to identify and classify the various abnormalities.
